# Asthma, Airway Symptoms and Rhinitis in Office Workers in Malaysia: Associations with House Dust Mite (HDM) Allergy, Cat Allergy and Levels of House Dust Mite Allergens in Office Dust

**DOI:** 10.1371/journal.pone.0124905

**Published:** 2015-04-29

**Authors:** Fang Lee Lim, Zailina Hashim, Leslie Thian Lung Than, Salmiah Md Said, Jamal Hisham Hashim, Dan Norbäck

**Affiliations:** 1 Department of Environmental and Occupational Health, Faculty of Medicine and Health Sciences, Universiti Putra Malaysia, UPM, Serdang, Selangor, Malaysia; 2 Department of Medical Microbiology and Parasitology, Faculty of Medicine and Health Sciences, Universiti Putra Malaysia, UPM, Serdang, Selangor, Malaysia; 3 Department of Community Health, Faculty of Medicine and Health Sciences, Universiti Putra Malaysia, UPM, Serdang, Selangor, Malaysia; 4 United Nations University-International Institute for Global Health (UNU-IIGH), Kuala Lumpur, Malaysia; 5 Department of Community Health, National University of Malaysia, Kuala Lumpur, Malaysia; 6 Uppsala University, Department of Medical Science, Occupational and Environmental Medicine, University Hospital, Uppsala, Sweden; Peking University, CHINA

## Abstract

A prevalence study was conducted among office workers in Malaysia (N= 695). The aim of this study was to examine associations between asthma, airway symptoms, rhinitis and house dust mites (HDM) and cat allergy and HDM levels in office dust. Medical data was collected by a questionnaire. Skin prick tests were performed for HDM allergens (*Dermatophagoides pteronyssinus*, *Dermatophagoides farinae)* and cat allergen *Felis domesticus*. Indoor temperature and relative air humidity (RH) were measured in the offices and vacuumed dust samples were analyzed for HDM allergens. The prevalence of *D*. *pteronyssinus*, *D*. *farinae* and cat allergy were 50.3%, 49.0% and 25.5% respectively. Totally 9.6% had doctor-diagnosed asthma, 15.5% had current wheeze and 53.0% had current rhinitis. The Der p 1 (from *D*. *pteronyssinus*) and Der f 1 (from *D*. *farinae*) allergens levels in dust were 556 ng/g and 658 ng/g respectively. Statistical analysis was conducted by multilevel logistic regression, adjusting for age, gender, current smoking, HDM or cat allergy, home dampness and recent indoor painting at home. Office workers with HDM allergy had more wheeze (p= 0.035), any airway symptoms (p= 0.032), doctor-diagnosed asthma (p= 0.005), current asthma (p= 0.007), current rhinitis (p= 0.021) and rhinoconjuctivitis (p< 0.001). Cat allergy was associated with wheeze (p= 0.021), wheeze when not having a cold (p= 0.033), any airway symptoms (p= 0.034), doctor-diagnosed asthma (p= 0.010), current asthma (p= 0.020) and nasal allergy medication (p= 0.042). Der f 1 level in dust was associated with daytime breathlessness (p= 0.033) especially among those with HDM allergy. Der f 1 levels were correlated with indoor temperature (p< 0.001) and inversely correlated with RH (p< 0.001). In conclusion, HDM and cat allergies were common and independently associated with asthma, airway symptoms and rhinitis. Der f 1 allergen can be a risk factor for daytime breathlessness.

## Introduction

Malaysia is a tropical country with warm and humid climate throughout the year. Continuous warm and humid environment will facilitate the growth and proliferation of both house dust mites (HDM) and storage mites. The mites have the ability to build up large populations as they can complete at least 12 life cycles per year [1]. One Malaysian study showed that *Blomia tropicalis* (8,934 mites/g), *Dermatophagoides pteronyssinus* (4,553 mites/g) and *Malayoglyphus intermedius* (1,020 mites/g) were three most common in home dust [2], while in Malaysian schools, levels of HDM (Der p 1 and Der f 1) and *Blomia tropicalis* (Blo t) allergens in school dust were low [3]. Clinical studies in patients demonstrated that allergy to HDM and cat allergens were the most common allergies in Malaysia [4–7]. However, we found no previous population based epidemiological study from Malaysia investigating associations between HDM allergens in office dust and the prevalence of asthma, airway symptoms and rhinitis in office workers.

Asthma and rhinitis are common respiratory diseases which are increasing globally [8,9], especially among the younger population in Asian cities [8]. The prevalence of rhinitis can be up to 45% in children and adolescents in low and middle-income countries in Asia [10]. However, there is a large variation of asthma among adults between Asian countries, the prevalence ranging from 0.7% to 11.9% [11]. One Asia-Pacific study reported that the average prevalence of allergic rhinitis was 8.7% among both children and adults [12]. The prevalence of doctor-diagnosed allergic rhinitis among children and adults ranged from 2.5% in Philipines to 12.3% in Vietnam [12]. These large variation indicates that environmental factors could play a role in the occurrence of asthma and rhinitis in Asia.

Sensitization to HDM and cat allergens may cause asthma [13–17] and rhinitis [18,19]. HDM allergens are a major indoor risk factor for asthma [20] triggering respiratory symptoms in many Asia countries [4,6,21–23]. Most epidemiology studies on HDM and cat allergens are from home environments [24,25] and few studies are available on allergens levels in office dust [26,27]. Some office environment studies from temperate climate have found low levels of HDM in office dust [28,29]. One study from the US reported a positive association between levels of HDM allergens in office dust and respiratory symptoms in office workers [30].

In a post-industrial society, a large proportion of the working population have office work. Since the 1980s there has been a focus on health effects due to the indoor environment in offices in the USA [31,32] and Europe [33]. However, there are few studies on associations between indoor environment in offices and asthma or rhinitis [34]. Respiratory illness among office workers has economical implications since they can increase sickness absentism and lost work time as well as decrease of quality of life [35]. Nevertheless, we found few epidemiological studies on associations between the office environment and respiratory health among office workers in tropical countries [36–38].

Carpets in offices can act as a reservoir of particle pollutants, including mites and mite allergens [39]. One study reported that offices with wall-to-wall carpet floors may contain HDM allergen in the carpets [26]. Offices with carpets have reported to have an excess of respiratory symptoms among the office workers [40–42] as well as impaired work performance and productivity [43]. These studies indicate that carpeting in offices can cause impaired respiratory health in office workers. However, no previous office study was found on respiratory health and HDM allergen levels in relation to carpeting in tropical countries.

The warm and humid conditions in tropics could provide suitable growth conditions for house dust mites in the office environment, especially if there are carpets. Thus, this study has investigated associations between selected office indoor environmental exposures; personal factors such as age, gender, current tobacco smoke status, HDM and cat allergy; and the respiratory health of office workers. The first aim was to measure the prevalence of sensitization to HDM and cat allergens, asthma, airway symptoms and rhinitis among office workers in an academic institution in Malaysia. The second aim was to study associations between personal factors and the health variables mentioned above. The third aim was to determine the HDM allergens levels in settled office dust, indoor temperature, relative air humidity (RH) and carbon dioxide (CO_2_) levels in the offices and the associations between wall-to-wall carpets and HDM allergens dust concentrations with the health variables mentioned above.

## Materials and Methods

### Study Population

The administrative office of the institution (UPM) provided a list of 61 offices in total. Administrative offices equipped with air-conditioning mechanical ventilation system with at least 15 office workers in one office were selected. Among the 61 offices listed, 50 fulfilled this inclusion criteria. However, four offices were excluded because the work tasks were different from ordinary office work. A letter was sent to the management of the 46 offices for study approval and 40 offices granted written or verbal permission. Thus, our study included 40 offices.

There were totally 2192 workers employed in the 40 offices selected and 701 workers agreed to participate (32.0%). All administrative workers aged 18 to 60 years were invited through emails or announcements by office representatives a few days before the data collection day. On the data collection day, the workers were invited again through face-to-face invitations. Six workers were excluded due to pregnancy thereby leaving 695 participants. A self-administered questionnaire was distributed to the respondents and collected within two weeks. The respondents had no information on the environmental data being collected from the offices when answering the questionnaires. The filled in questionnaires were reviewed and respondents were consulted if there were any uncertainty in the information provided. Among the 695 participants, two were excluded from skin prick test due to recent severe asthma leaving 463 workers (66.7%) participating in the skin prick test.

Potential selection bias was investigated by interviewing non-participants at the end of the study. One to two randomly selected workers from each office, who did not participate in the questionnaire study, answered four yes/no health questions (ever doctor-diagnosed asthma, ever allergic rhinitis, rhinitis in the past 12 months and ever eczema) in a face-to-face interview. These interview questions were exactly the same questions as in the questionnaire study.

### Questionnaire

The questionnaire was adapted from the European Community Respiratory Health Survey (ECRHS) [44] and the International Study of Asthma and Allergies in Childhood (ISAAC) [45]. It contained questions on the history of asthma, as well as airway and rhinitis symptoms. Respiratory symptoms in this study were defined as asthma, airway or rhinitis symptoms. The questionnaire consisted of four sections. The sections are described bellows:

#### Questions on personal factors and the home environment

1. Gender (male/female);
2. Age;
3. Ethnicity (Malay/Chinese/Indian/Others);
4. What is your tobacco smoking status? (Never smoked/former smoker/current smoker);
5. Are there any pets at your dwelling? (yes/no);
  - If “Yes”, what type of pet? (dog/cat/bird/fish/others);
6. Have the interior of your dwelling been painted during the last 12 months? (yes/no);
7. Have any of the following been identified in your dwelling during the last 12 months?
  - Water leakage or water damage indoors in walls, floor or ceiling (yes/no);
  - Bubbles or yellow discoloration on plastic floor covering or black discoloration on parquet floor (yes/no);
  - Visible mould growth on indoors on walls, floor or ceilings (yes/no);
  - The smell of mould in one or more rooms (not the basement) (yes/no);

#### Questions on airway symptoms

1. Have you had wheezing or whistling in the chest at any time in the last 12 months? (yes/no);
2. Have you been at all breathless when the wheezing noise was present? (yes/no);
3. Have you had this wheezing or whistling when you did not have a cold? (yes/no);
4. Have you had an attack of shortness of breath that came on during the day when being at rest at any time in the last 12 months? (yes/no);
5. Have you had an attack of shortness of breath that came on following strenuous activity at any time in the last 12 months? (yes/no);
6. Have you ever been woken by an attack of shortness of breath in the last 12 months? (yes/no);

#### Questions on asthma

1. Have you ever had asthma? (yes/no);
2. Was the asthma diagnosed by a physician? (yes/no);
3. Have you had any attack of asthma in the last 12 months? (yes/no);
4. Are you currently using any asthma medication? (spray, inhalation powders, tablets) (yes/no);

#### Questions on rhinitis

1. Do you have any nasal allergies, including hay fever? (yes/no);
2. Have you ever had a problem with sneezing, or a runny or a blocked nose when you did not have a cold or the flu in the last 12 months? (yes/no);
3. Has this nose problem been accompanied by itchy or watery eyes? (yes/no);
4. Have you used any steroid nasal sprays, antihistamines pills, capsules or tablets for treatment of your nasal disorder in the last 12 months? (yes/no);

### Skin prick test

The office workers were informed on the procedure for the skin prick test one week before the testing. In addition, they were advised to avoid using any antihistamine drug until they had completed the test. Pregnant women (n = 6), subjects on beta-blocker medication, with severe dermatographism or persistent severe or unstable asthma (n = 2) were excluded from testing to avoid risk of anaphylaxis or difficulties in interpretation of test result [46].

The skin prick tests were carried out by experienced hospital nurses. Workers had to share information about their recent medication intake before the skin prick test was performed. Skin prick test kits (ALK Abello SA, Madrid, Spain) for *D*. *pteronyssinus*, *D*. *farinae* and *Felis domesticus* were used. The concentration of *D*. *pteronyssinus* and *D*. *farinae* allergen extracts were 30 HEP (histamine equivalent prick) while *Felis domesticus* allergen extract was 10 HEP. Normal saline solution and histamine were used as negative and positive controls. After 20 minutes, the wheal diameter was measured by adding the largest diameter and its perpendicular diameter and dividing the sum with two [22]. The mean wheal diameter for each control and allergen were calculated. The test result was considered as positive if the mean wheal diameter was ≥ 3 mm.

### Building inspection and climate measurement

Due to time constrains, 25 offices out of the 40 were randomly selected for building inspection, as well as indoor and outdoor climate measurements. Then 15 offices (60%) which had 15 to 55 office workers were selected from the 25. Subsequently 10 offices (66%) with more than 55 office workers were then selected from 15 offices. In each of these 25 offices, one to five administrative rooms were included in the environmental measurements, depending on the size of the office. All rooms involved in dust sample collection were inspected and the indoor climate was measured.

A building inspection was carried out before indoor climate measurements and dust samplings were obtained. Signs of dampness such as water leakage, indoor mould growth, odor in the offices, information on the building materials, floor furnish, types of ventilation systems and presence of indoor plants in the office room were recorded. Questions were asked on the frequency of office cleaning and maintenance of the mechanical ventilation systems. A Q-trak indoor air quality monitor 7565 (TSI Incorporated, St. Paul, MN, USA) was used to measure indoor temperature (°C), RH (%), CO_2_ concentration (ppm) and carbon monoxide (CO) (ppm) during normal activities in the center of room. The Q-trak monitor was placed at height 1–1.5 m above the floor level and around 1 m from the workers for 50–70 minutes by logging average values per minute [47]. There was one Q-trak measurement for each office room during the dust sample collection, and two large offices had two Q-trak measurements. The outdoor climate was measured by the Q-trak instrument outside the office building on the same day.

### Dust collection

In each of the 25 offices, dust was collected from one to five administrative rooms (2–10 dust samples), depending on the size of the office. Dust samples were collected from the main administrative rooms of each office. Each room was divided into two parts (entrance side half and window side half) and a dust sample was collected from each part [48]. Settled dust on floor, tables and chairs was collected by using a 1800 W electric power vacuum cleaner (Model PVC-31A, Pensonic, Malaysia) equipped with a special dust collector fitted with a Millipore filter (pore size 6 μm) (ALK Abello, Copenhagen, Denmark). The dust samples were collected by vacuum cleaning for eight minutes per sample, four minutes vacuuming of floor and four minutes vacuuming of tables/ chairs. The settled dust samples were sieved (particle diameter < 400 μm) to obtain fine dust [47]. The sieved dust samples was weighted and stored at -20°C until extraction [48].

### Extraction and analysis of dust mites’ allergen

For extraction of house dust mite allergens, 2 ml of phosphate-buffer saline with Tween (PBS-T) was added into each fine dust sample (100 ± 5 mg) in a centrifuge tube. The centrifuge tubes were shaken for two hours at room temperature and centrifuged for 10 minutes at 3000 rpm [49]. Then, the supernatants were transferred into Eppendorf tube and stored at -20°C until analysis of dust mites. Two-sites monoclonal antibody (MAB) sandwich ELISAs (Indoor Biotechnologies, Inc., Charlottesville, VA) were used to analyze for Der p 1 and Der f 1. Analysis were performed according to the manufacturer’s protocols with the exception that Streptavidin-horseradish peroxidase was obtained from Nacalai Tesque, Inc., Kyoto, Japan. All units were expressed as ng/g [50]. The detection limit for HDM allergens was 10 ng/g.

### Statistical analysis

All statistical tests were performed by the Statistical Package for the Social Sciences (SPSS) 21.0 or the STATA statistical package 11.0 (for multi-level logistic regression). Initially, associations between personal factors (age, gender, smoking status, HDM allergy and cat allergy), asthma and respiratory symptoms were analyzed by Chi-square test. The symptom differences between participants and non-participant groups in the questionnaire study, skin prick test and environmental sampling study were also examined by Chi-square test.

The Der p 1 and Der f 1 level from dust samples were not normally distributed, thus median, interquartile range, minimum and maximum were reported and non-parametric statistical tests were used. Kendall’s tau beta test was applied to study the correlations between Der p 1 and Der f 1 levels in office dust; and environmental variables (indoor temperature, RH, CO_2_, CO and amount of sieved dust). Mann Whitney-U test was applied to test the differences between offices with and without carpeting, amount of dust and allergen level. Chi-square test was used to study associations between floor carpeting and respiratory symptoms.

Finally, associations between risk factors and symptoms were studied by two-level multiple logistic regression (individual level and office level) with each model including personal risk factors such as age, gender, smoking status, HDM and cat allergy. The associations between level of allergens in office dust samples and floor carpeting were analyzed with adjustment for age, gender, smoking status, HDM and cat allergy, any home dampness and indoor home painting in the last 12 months. Stratified analysis (stratified for HDM allergy) was performed using the same models. Two-tailed tests and a 5% level of significance were applied in all statistical analysis.

### Ethics Statement

The study was approved by the Ethics Committee for Research Involving Human Subjects in Universiti Putra Malaysia (UPM). Written consents were obtained from all respondents after the briefing on the study objective and procedures. For data collection on the study location, permission was obtained from the authorities.

## Result

A total of 695 workers participated in the study and 66.6% were females. The mean age was 34 years (range 18–59 years), with a majority of Malay (97.8%), followed by Chinese (0.9%). Overall, there were higher prevalence of current airway symptoms and asthma among females as compared to males (Table 1). Breathlessness during wheeze, daytime attack of breathlessness at rest, any daytime breathlessness, doctor-diagnosed asthma, current asthma medication, current asthma attack and current asthma were significantly higher among females. However, there was no significant difference in airway, asthma and rhinitis symptoms between smokers and non-smokers. A total of 30.2% of the men and 0.4% of the women were current smokers (p < 0.001).

**Table 1:** Asthma and respiratory symptoms among office workers, stratified for gender and smoking (n = 695).

Respiratory symptoms		Male (n = 232), n (%)	Female (n = 463), n (%)	OR	95% CI	p-value	Non-smoker (n = 619), n (%)	Smoker (n = 72), n (%)	OR	95% CI	p-value	Total (n = 695), n (%)
**Questions on current airway symptoms**	**Wheeze or whistling in the chest**	29 (12.6)	78 (17.0)	1.42	0.90 – 2.25	0.148	97 (15.7)	10 (13.9)	0.87	0.43 – 1.75	0.863	107 (15.5)
	**Breathlessness during wheeze**	14 (6.1)	56 (12.2)	2.15	1.17–3.95	**0.011** *	65 (10.5)	5 (6.9)	0.64	0.25 – 1.64	0.415	70 (10.1)
	**Wheezing or whistling when did not have cold**	11 (4.8)	34 (7.4)	1.60	0.79 – 3.21	0.252	40 (6.5)	5 (6.9)	1.08	0.41 – 2.83	0.875	45 (6.5)
	**Daytime attack of breathlessness at rest**	13 (5.7)	58 (12.6)	2.40	1.28 – 4.47	**0.005** **	67 (10.8)	4 (5.6)	0.49	0.17 – 1.37	0.165	71 (10.3)
	**Daytime attack of breathlessness after strenuous activity**	25 (10.8)	79 (17.2)	1.72	1.07 – 2.78	**0.032** *	95 (15.3)	9 (12.5)	0.79	0.38 – 1.64	0.604	104 (15.1)
	**Any daytime breathlessness**	29 (12.5)	99 (21.4)	1.90	1.22 – 2.98	**0.005** **	118 (18.9)	10 (13.9)	0.69	0.34 – 1.39	0.339	128 (18.4)
	**Nocturnal attacks of breathlessness**	18 (7.8)	56 (12.2)	1.64	0.94 – 2.87	0.090	67 (10.8)	7 (9.7)	0.89	0.39 – 2.01	1.000	74 (10.7)
	**At least one airway symptoms** ^**a**^	51 (22.0)	133 (28.7)	1.43	0.99 – 2.07	0.068	168 (27.0)	16 (22.2)	0.77	0.43 – 1.39	0.481	184 (26.5)
**Questions on asthma**	**Ever asthma**	22 (9.5)	63 (13.6)	1.50	0.90–2.51	0.140	80 (12.8)	5 (6.9)	0.51	0.20 – 1.30	0.184	85 (12.2)
	**Doctor-diagnosed asthma**	13 (5.6)	54 (11.7)	2.22	1.19 – 4.17	**0.010** *	64 (10.3)	3 (4.2)	0.38	0.12 – 1.24	0.136	67 (9.6)
	**Current asthma medication**	11 (4.7)	45 (9.7)	2.16	1.10 – 4.27	**0.026** *	53 (8.5)	3 (4.2)	0.47	0.14 – 1.54	0.256	56 (8.1)
	**Current asthma attacks**	8 (3.4)	41 (8.9)	2.72	1.25 – 5.90	**0.007** **	46 (7.4)	3 (4.2)	0.55	0.17 – 1.80	0.464	49 (7.1)
	**Current asthma** ^**b**^	12 (5.2)	49 (10.6)	2.17	1.13 – 4.17	**0.022** *	58 (9.3)	3 (4.2)	0.42	0.13 – 1.39	0.187	61 (8.8)
**Questions on rhinitis**	**Ever allergic rhinitis**	56 (24.1)	98 (21.2)	0.84	0.58 – 1.23	0.384	137 (22.0)	17 (23.6)	1.10	0.62 – 1.95	0.765	154 (22.2)
	**Rhinitis in the last 12 months**	134 (57.8)	234 (50.5)	0.75	0.54 – 1.03	0.077	325 (52.2)	43 (59.7)	1.36	0.83 – 2.23	0.262	368 (53.0)
	**Rhinoconjuctivitis in the last 12 months**	76 (32.8)	162 (35.0)	1.11	0.79 – 1.54	0.611	212 (34.1)	26 (36.1)	1.09	0.66 – 1.82	0.793	238 (34.3)
	**Nasal allergies medication in the last 12 months**	30 (12.9)	55 (11.9)	0.91	0.56 – 1.46	0.713	73 (11.7)	12 (16.7)	1.51	0.77 – 2.93	0.252	85 (12.2)

^a^Wheeze or whistling in the chest, daytime attacks of breathless at rest or at exercise, or nocturnal attacks of breathlessness during the last 12 months.

^b^Current asthma medication or asthma attacks during the last 12 months.

*p<0.05

**p<0.01

**Fig 1:**
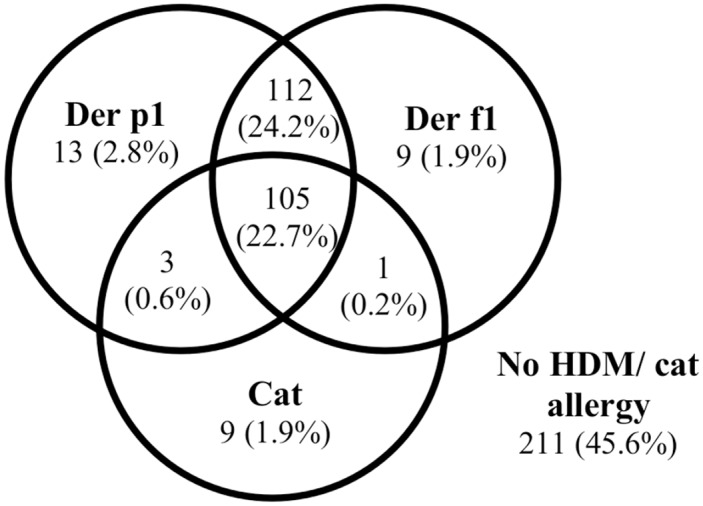
Venn diagram for the distribution of subjects with a positive skin prick test to *D*. *pteronyssinus*, *D*. *farinae* and *Felis domesticus* in the group with skin prick test (n = 463).

We compared prevalence of asthma, rhinitis and eczema among participants and non-participants groups in the questionnaire study, the skin prick test, and the environment measurement. There were no significant differences between participants and non-participants (Table 2). A total of 16 office workers (3.5%) were sensitized only to Der p 1, 10 (2.2%) were sensitized only to Der f 1, and 217 (52.5%) were sensitized to Der p 1 or Der f 1. Many respondents were sensitized to cat allergy, but most of them were sensitized to both HDM and cat allergy (Fig 1). Participants with HDM allergy or cat allergy had significantly higher prevalence of asthma and respiratory symptoms (Table 3). In this study, 19.9% had cats in their current home. Those with cats at home had more daytime breathlessness at rest (OR = 1.81, 95%CI 1.05–3.13, p = 0.041) but there was no significant association between cat keeping and other types of respiratory symptoms. Moreover, there was no association between cat keeping and cat allergy (OR = 1.01, 95%CI 0.59–1.70, p = 1.000).

**Table 2:** Comparison of asthma, rhinitis and eczema among participants and non-participants.

Respiratory symptoms	Questionnaire			Skin prick test			Environmental data		
	Participants (n = 695), n (%)	Non-participants^a^ (n = 60), n (%)	p-value^b^	Participants (n = 462), n (%)	Non-participants (n = 233), n (%)	p-value^b^	Participants with env. data (n = 506), n (%)	Participants without env. data (n = 189), n (%)	p-value^b^
**Ever doctor diagnosed asthma**	67 (9.6)	10 (16.7)	0.12	44 (9.5)	23 (9.9)	0.89	43 (8.5)	24 (12.7)	0.11
**Ever allergic rhinitis**	154 (22.2)	16 (26.7)	0.42	44 (18.9)	110 (23.8)	0.15	119 (23.5)	35 (18.5)	0.18
**Rhinitis in the past 12 months**	368 (53.0)	28 (46.7)	0.35	256 (55.5)	112 (48.1)	0.07	269 (53.3)	99 (52.4)	0.87
**Ever eczema**	219 (31.5)	15 (25.0)	0.38	149 (32.3)	70 (30.0)	0.60	166 (32.8)	53 (28.0)	0.24

^a^ Non-participants (one to two per office) were interviewed for selective questions

^b^ Comparison by using Chi-square test for four field contingency tables

**Table 3:** Asthma and respiratory symptoms among office workers with skin prick test (n = 463), stratified for house dust mite (HDM) and cat allergy.

Respiratory symptoms		No HDM allergy (n = 220), n (%)	Any HDM allergy (n = 243),n (%)	OR	95% CI	p-value	No cat allergy (n = 345), n (%)	Cat Allergy (n = 118), n (%)	OR	95% CI	p-value	Total (n = 463), n (%)
**Questions on current airway symptoms**	**Wheeze or whistling in the chest**	24 (10.9)	54 (22.6)	2.38	1.42 – 4.01	0.001**	46 (13.4)	32 (27.8)	2.50	1.50 – 4.17	0.001**	78 (17.0)
	**Breathlessness during wheeze**	13 (5.9)	38 (15.9)	3.01	1.56 – 5.82	0.001**	29 (8.4)	22 (19.1)	2.57	1.41–4.68	**0.003** **	51 (11.1)
	**Wheezing or whistling when did not have cold**	10 (4.5)	24 (10.0)	2.34	1.09 – 5.02	**0.031** *	18 (5.2)	16 (13.9)	2.93	1.44 – 5.95	**0.004** **	34 (7.4)
	**Daytime attack of breathlessness at rest**	21 (9.5)	33 (13.7)	1.49	0.84 – 2.69	0.193	35 (10.2)	19 (16.2)	1.71	0.94 – 3.13	0.095	54 (11.7)
	**Daytime attack of breathlessness after strenuous activity**	26 (11.8)	47 (19.4)	1.80	1.07 – 3.02	**0.030** *	49 (14.2)	24 (20.5)	1.56	0.91 – 2.68	0.109	73 (15.8)
	**Any daytime breathlessness**	34 (15.5)	56 (23.0)	1.64	1.02 – 2.63	**0.046** *	60 (17.4)	30 (25.4)	1.62	0.98 – 2.67	0.060	90 (19.4)
	**Nocturnal attacks of breathlessness**	19 (8.7)	33 (13.6)	1.66	0.92 – 3.02	0.106	36 (10.5)	16 (13.7)	1.36	0.72 – 2.55	0.397	52 (11.3)
	**At least one airway symptoms** ^**a**^	46 (20.9)	84 (34.6)	2.00	1.31 – 3.04	**0.001** **	82 (23.8)	48 (40.7)	2.20	1.41 – 3.43	**0.001** **	130 (28.1)
**Questions on asthma**	**Ever asthma**	12 (5.5)	45 (18.5)	3.94	2.02– 7.67	**<0.001** ***	28 (8.1)	29 (24.6)	3.69	2.09 – 6.52	**<0.001** ***	57 (12.3)
	**Doctor-diagnosed asthma**	8 (3.6)	37 (15.2)	4.76	2.17–10.47	**<0.001** ***	21 (6.1)	24 (20.3)	3.94	2.10 – 7.39	**<0.001** ***	45 (9.7)
	**Current asthma medication**	6 (2.7)	24 (9.9)	3.91	1.57–9.75	**0.002** **	15 (4.3)	19 (16.1)	4.22	2.07 – 8.62	**<0.001** ***	34 (7.3)
	**Current asthma attacks**	6 (2.7)	24 (9.9)	3.91	1.57–9.75	**0.002** **	14 (4.1)	16 (13.6)	3.71	1.75 – 7.86	**0.001** **	30 (6.5)
	**Current asthma** ^**b**^	6 (2.7)	31 (12.8)	5.22	2.13–12.76	**<0.001** ***	17 (4.9)	20 (16.9)	3.94	1.99 – 7.81	**<0.001** ***	37 (8.0)
**Questions on nasal allergies**	**Ever allergic rhinitis**	27 (12.3)	84 (34.6)	3.78	2.33 – 6.11	**<0.001** ***	58 (16.8)	53 (44.9)	4.04	2.55–6.39	**<0.001** ***	111 (24.0)
	**Rhinitis in the past 12 months**	102 (46.4)	155 (63.8)	2.04	1.40–2.96	**<0.001** ***	176 (51.0)	81 (68.6)	2.09	1.34 – 3.25	**0.001** **	257 (55.5)
	**Rhinoconjuctivitis in the past 12 months**	51 (23.2)	113 (46.5)	2.88	1.93 – 4.31	**<0.001** ***	107 (31.0)	57 (48.7)	2.11	1.38 – 3.24	**0.001** **	164 (35.5)
	**Nasal allergies medication in the past 12 months**	18 (8.2)	44 (18.1)	2.48	1.39 – 4.44	**0.002** **	35 (10.1)	27 (22.9)	2.63	1.51 – 4.57	**0.001** **	62 (13.4)

^a^Wheeze or whistling in the chest, daytime attacks of breathless at rest or at exercise, or nocturnal attacks of breathlessness during the last 12 months.

^b^Current asthma medication or asthma attacks during the last 12 month

*p<0.05;

**p<0.01;

***p<0.001

All 35 office rooms in the 25 offices were in concrete buildings with mechanical ventilation system. Most of the rooms had split unit air conditioning systems, seven rooms had centralized air conditioning systems and eight rooms had a combination of centralized and split unit air conditioning systems. Among these 35 office rooms, three had visible indoor mould growth and seven had signs of water leakage. In total, 15 office rooms had a wall-to-wall carpet, 17 had tiles (stone materials), two had PVC (Polyvinyl chloride) plastic and one had a wooden floor.

Table 4 shows levels of Der p 1 and Der f 1 in office dust samples and the amount of sieved dust from the sampling filters. Although both allergens had similar median concentrations, some dust samples had much higher level of Der f 1 allergens. There was a tendency of an inverse correlation between Der p 1 and Der f 1 levels (p = 0.068). We examined the correlations between HDM allergens levels and indoor environmental variables. Der f 1 levels were positively correlated with indoor temperature (p < 0.001) and negatively correlated with RH (p < 0.001).

**Table 4:** Office environment and HDM allergens in the dust samples of 35 office rooms.

Environmental Parameters	Median	Interquartile range (25 percentile -75 percentile)	Minimum-maximum
**Level of Der p1 (ng/g) (n = 70)**	556.2	376.2 – 581.2	267.0 – 935.4
**Level of Der f1 (ng/g) (n = 70)**	658.0	421.3 – 1135.8	174.0 – 14107.0
**Amount of sieved dust (g)(n = 70)**	1.0	0.6 – 1.2	0.1 – 2.4

In addition, we studied associations between floor carpeting, and amount of sieved dust and allergen levels. There was no significant difference between office rooms with carpet and non-carpet floors for Der p 1 allergen level (floor carpeting with median Der p 1 = 467.7 ng/g, IQR = 375.4–581.8; and non-carpet floors with median Der p 1 = 568.7 ng/g, IQR = 380.5–581.1; p = 0.094) or Der f 1 allergen level (floor carpeting with median Der f 1 = 795.6 ng/g, IQR = 450.6–1118.5; and non-carpet floors with median Der f 1 = 516.0 ng/g, IQR = 395.0–1164.1; p = 0.431). Moreover, there was no significant difference in the amount of sieved dust in carpeted rooms (median = 0.99 g, IQR = 0.76–1.23) as compared to non-carpeted rooms (median = 0.91 g, IQR = 0.49–1.124; p = 0.520).

As a next step, we investigated associations between floor carpeting, HDM allergens levels and respiratory symptoms in bivariate analysis. There was an inverse association between wall-to-wall carpeting and wheezing when not having a cold (OR = 0.37, 95%CI 0.16–0.89, p = 0.025). However, no other respiratory symptoms were significantly associated with wall-to-wall carpets.

Associations between asthma, respiratory symptoms, personal factors and home environment factors were analyzed using multilevel logistic regression model (two levels, subject and office), including age, gender, current smoking, HDM allergy, cat allergy, any home dampness and indoor home painting in the last 12 months in the model (Table 5). Younger office workers had more rhinitis and rhinoconjuctivitis. Females had more wheeze, wheezing when not having a cold, daytime attack of breathlessness, nocturnal attacks of breathlessness and doctor-diagnosed asthma. Finally, those with HDM allergy had more wheeze, at least one airway symptoms, doctor-diagnosed asthma, current asthma, rhinitis and rhinoconjuctivitis. Finally, those with cat allergy had more wheeze, wheeze when not having a cold, at least one airway symptom, doctor-diagnosed asthma, current asthma and nasal allergy medication.

**Table 5:** Associations between asthma, respiratory symptoms, personal factors and home environment factors among office workers (n = 461) [OR(95%CI)].

Variables	Wheeze or whistling in the chest	Wheezing or whistling when did not have cold	Any daytime breathlessness	Nocturnal attacks of breathlessness	At least one airway symptoms^a^	Doctor-diagnosed asthma	Current asthma^b^	Rhinitis in the past 12 months	Rhino-conjuctivitis	Nasal allergies medication in the past 12 months
**Age** ^**c**^	1.07	0.94	0.81	0.79	0.88	0.95	1.06	**0.72**	**0.69**	0.92
	(0.81–1.40)	(0.62–1.41)	(0.61–1.07)	(0.56–1.12)	(0.69–1.11)	(0.66–1.37)	(0.73–1.55)	**(0.`58–0.88)** **	**(0.54–0.87)** **	(0.67–1.27)
**Gender**	**1.88**	**3.15**	**2.33**	**2.98**	1.60	**2.41**	2.08	1.03	1.38	1.23
	**(1.00–3.55)** *	**(1.13–8.81)** *	**(1.26–4.33)** *	**(1.27–7.01)** *	(0.96–2.66)	**(1.07–5.40)** *	(0.88–4.51)	(0.65–1.63)	(0.85–2.26)	(0.62–2.43)
**Current Smoking**	1.25	2.30	1.27	2.19	1.08	0.88	1.00	1.27	1.28	1.55
	(0.49–3.50)	(0.57–9.23)	(0.50–3.24)	(0.69–6.94)	(0.50–2.35)	(0.22–3.49)	(0.25–4.03)	(0.63–2.53)	(0.63–2.61)	(0.60–3.97)
**HDM allergy**	**1.91**	1.69	1.63	1.77	**1.70**	**3.54**	**3.84**	**1.66**	**2.75**	1.97
	**(1.05–3.50)** *	(0.69–4.15)	(0.94–2.83)	(0.90–3.50)	**(1.05–2.76)** *	**(1.47–8.52)** **	**(1.43–10.30)** **	**(1.08–2.56)** *	**(1.73–4.37)** ***	(0.99–3.91)
**Cat allergy**	**2.06**	**2.59**	1.36	1.08	**1.74**	**2.55**	**2.49**	1.52	1.25	**1.97**
	**(1.12–3.79)** *	**(1.08–6.22)** *	(0.76–2.43)	(0.53–2.21)	**(1.04–2.90)** *	**(1.25–5.22)** *	**(1.16–5.38)** *	(0.92–2.53)	(0.76–2.06)	**(1.02–3.79)** *

*p-value<0.05

**p-value<0.01

***p-value<0.001

^a^Wheeze or whistling in the chest, daytime attacks of breathless at rest or at exercise, or nocturnal attacks of breathlessness during the last 12 months.

^b^Current asthma medication or asthma attacks during the last 12 month

^c^OR calculated for 10 year increase in age

Each mutual adjustment model was analyzed by two-level logistic regression keeping age, gender, current smoking status, HDM allergy, cat allergy, any home dampness, indoor home painting in the last 12 months in the models.

In bivariate analysis, level of Der f 1 allergen was not significantly associated with current airway symptoms, asthma and allergic rhinitis but Der p1 allergen level was inversely associated with wheeze (p = 0.025) and at least one airway symptom (p = 0.014). We applied multilevel logistic regression analysis (two levels, subject and office), to analyze associations between levels of Der p 1, Der f 1, office floor carpeting and the 10 asthma and respiratory symptoms, adjusting for age, gender, current smoking status, HDM allergy, cat allergy, any home dampness and indoor home painting in the last 12 months (Table 6). Level of Der p 1 was negatively associated with wheeze and at least one airway symptoms. In contrast, level of Der f 1 was positively associated with any daytime breathlessness. We also analyzed the significant associations stratifying for HDM allergy. The negative association between wheeze and Der p 1 level was stronger among those with no HDM allergy (OR = 0.61, 95%CI 0.35–1.06, p = 0.082) as compared to those with HDM allergy (OR = 0.74, 95%CI 0.52–1.07, p = 0.110). The inverse association between at least one airway symptoms and the Der p 1 level was significant among those with HDM allergy (OR = 0.73, 95%CI 0.54–1.00, p = 0.054), but not among those without HDM allergy (OR = 0.71, 95%CI 0.47–1.09, p = 0.117). Finally, the positive association between any daytime breathlessness and the Der f 1 level was significant among those with HDM allergy (OR = 1.16, 95%CI 1.01–1.32, p = 0.033) but not among those without HDM allergy (OR = 1.03, 95%CI 0.79–1.35, p = 0.809).

**Table 6:** Associations between asthma, respiratory symptoms, levels of Der p 1 and Der f 1in office dust samples and office floor carpeting among office workers (n = 371)[OR(95%CI)].

Variables	Wheeze or whistling in the chest	Wheezing or whistling when did not have cold	Any daytime breathlessness	Nocturnal attacks of breathlessness	At least one airway symptoms^a^	Doctor-diagnosed asthma	Current asthma^b^	Rhinitis in the past 12 months	Rhino-conjuctivitis	Nasal allergies medication in the past 12 months
**Level of Der p 1** ^**c**^	**0.69**	0.84	0.81	0.85	**0.72**	0.89	0.70	0.97	0.90	1.11
	**(0.51–0.93)** *	(0.53–1.34)	(0.61–1.08)	(0.60–1.21)	**(0.55–0.92)** **	(0.59–1.33)	(0.45–1.10)	(0.76–1.24)	(0.71–1.15)	(0.75–1.65)
**Level of Der f 1** ^**d**^	0.90	1.01	**1.12**	0.93	1.05	0.97	0.84	1.03	1.04	1.07
	(0.75–1.08)	(0.82–1.25)	**(1.00–1.25)** *	(0.77–1.14)	(0.95–1.17)	(0.80–1.19)	(0.60–1.19)	(0.92–1.15)	(0.93–1.15)	(0.92–1.25)
**Floor carpeting**	0.96	0.55	0.60	1.03	0.98	1.00	0.91	1.07	0.85	0.81
	(0.51–1.82)	(0.19–1.605)	(0.32–1.12)	(0.48–2.18)	(0.58–1.66)	(0.42–2.37)	(0.35–2.35)	(0.63–1.81)	(0.50–1.46)	(0.34–1.93)

*p-value<0.05

**p-value<0.01

^a^Wheeze or whistling in the chest, daytime attacks of breathless at rest or at exercise, or nocturnal attacks of breathlessness during the last 12 months.

^b^Current asthma medication or asthma attacks during the last 12 month

^c^OR calculated for 100 ng/g increase in level of Der p 1 allergen

^d^OR calculated for 1000 ng/g increase in level of Derf 1 allergen

Each model was analyzed by two-level logistic regression and adjusting for age, gender, current smoking status, HDM allergy, cat allergy, any home dampness and indoor home painting in the last 12 monthsin the models.

## Discussion

HDM and cat allergies were very common among the office workers in this study and these allergies were independently associated with asthma, respiratory symptoms and rhinitis. The majority of those with HDM allergy were sensitized to both Der p 1 and Der f 1. Der f 1 level in office dust was correlated with indoor temperature but inversely correlated with indoor relative air humidity and there was a positive association between Der f 1 levels in office dust and daytime breathlessness, especially in HDM sensitized subjects. One limitation of this study is that we did not include other allergens, such as cockroach and *Blomia tropicalis* (storage mites) in our skin prick test. Thus, we might underestimate the true prevalence of Ig E-mediated allergy among the office workers.

We invited all offices within a major academic institution in Klang Valley, Malaysia. The office rooms involved in environmental data sampling were randomly selected from the office list. Thus, bias due to selection of buildings or rooms is less likely. Since the participation rate was relatively low in this study, we collected data on respiratory health among a sub-sample of non-participants by conducting a short face-to-face interview using questions from the study questionnaire. We found no significant difference in respiratory health between respondents and non-respondents in the questionnaire study, in the allergy testing and in the environmental measurements.

Information bias can influence epidemiological studies. In this study, the respondents did not have any information on the results of environmental measurement or the clinical tests when they answered the questionnaire. The questionnaire was answered before the clinical investigation and the environmental measurements. Therefore, information bias is less likely. Environmental sampling was conducted within two weeks from the day when respondents answered the questionnaire and all the dust samples were analyzed after the questionnaire data collection was completed. The same calibrated instrument was used all measurements. Dust samples were analyzed in an arbitrary sequence at the laboratory without any knowledge about the health status of respondents or environmental measurements. All dust samples were analyzed by the same person using the same laboratory equipment.

The data was analyzed by both bivariate and multivariate statistical analysis, mostly with similar results. Thus, we believe that our conclusions were not seriously influenced by selection or information bias or choice of a particular statistical model. However, cross sectional study design limits the possibility to draw conclusions about causal relationships. The majority of respondents were Malays, and the study was performed in one major academic institution in the Klang Valley only. To our knowledge, our study is the first study of office workers in Malaysia using skin prick allergy testing. Further larger studies in different areas are needed to verify the high prevalence of respiratory symptoms and HDM and cat allergy among office workers in Malaysia.

The prevalence of doctor-diagnosed asthma (9.6%), current wheeze (15.5%) and current rhinitis (53.0%) was high among the office workers. According to the world health survey [51], the prevalence of doctor-diagnosed adult asthma in Malaysia was 5.2%. In Singapore, 5.1% of adults had doctor-diagnosed adult asthma [52] and in Thailand, the prevalence of doctor-diagnosed asthma was 11.6% [53]. Song and colleagues has reviewed adult asthma in Asia and found a large variation between countries. The prevalence of adult asthma ranged from 0.7% in rural areas outside Beijing (China) to 12.0% in Al Ain (United Arab Emirates) [11]. For the other respiratory symptoms, the prevalence of wheeze in the last 12 months among adults in Malaysia has been reported to be 7.55% [51] and the prevalence of allergic rhinitis in Malaysia has been reported to be 7.1% [12]. The prevalence of adult allergic rhinitis in the Asia-Pacific region ranges from 8.7%, with Australia on the top rank (13.2%) and followed by Vietnam (12.3%) and Taiwan (9.6%) [12]. The prevalence of respiratory symptoms in this study was higher than in many other studies from Asian countries, which may be due to the high prevalence of HDM and cat allergies among office workers in this study. Previous population based studies have showed significant associations between HDM or cat sensitization with higher prevalence of asthma and respiratory symptoms [54,55]. Moreover, respondents with HDM sensitization reported higher prevalence of nasal symptoms compared to respondents with no HDM sensitization [56].

One important risk factor for asthma and airway symptoms in our study was female gender. This is in agreement with some previous population studies from western countries, but gender differences in respiratory health in Asia seem to be more unclear. One reason could be that in many Asian countries, very few women are smokers while the prevalence of smoking is high among men. However in our study, the gender differences remained significant even after adjusting for smoking habits. A population study among adults in European Union countries reported that females have a higher incidence of asthma in adulthood [57]. A population based cohort study found that the incidence of non-allergic asthma was higher in women than men [58]. All available studies in gender differences in population studies from Asia are prevalence studies. One prevalence study from Ulaanbaatar, Mongolia, reported that women had slightly higher prevalence of doctor-diagnosed asthma, wheeze and allergic rhinitis [59]. In contrast, a study among university students in Phitsanulok, Thailand, found a lower prevalence of wheeze and asthma among females as compared to men [60]. One study from the northern part of Vietnam reported that males had more asthma, but they found no gender differences for allergic rhinitis [61]. One nationwide population survey in Thailand did not find any gender differences in respiratory symptoms [62].

In this study, the prevalence of cat allergy was high (25.4%) and 19.9% of respondents had a cat in their current dwelling. Cat allergy is a common allergy in some parts of Europe, Australia and New Zealand [44,63]. The overall prevalence of cat allergy in the 10-year follow up of the ECRHS (ECRHS II) was 7.8% [64]. However it seems that cat allergy has not been studied much in Asian countries, especially not among adults in South East Asia. One population study reported 4.1% cat allergy among children in Thailand [65]. Another study among adults in the northern part of Vietnam found 4.5% cat allergy [22] and one population study from China, including both children and adults, found 1.6% cat allergy [66]. Thus, the prevalence of cat allergy in our study was higher than previous studies on cat allergy from Asia.

There are also studies on cat allergy among hospital cases of patients with asthma and allergic rhinitis. Two studies among rhinitis patients in Malaysia found 20.0% [6] and 41.6% [7] of cat allergy respectively. One study among university staff and students in Thailand reported that 45.6% of the asthmatic subjects had cat allergy [53]. In Singapore, one study on rhinitis patients including both children and adults found 29.1% cat allergy [67] and another Singapore adult rhinitis patient study found 9.4% cat allergy [68]. One Hong Kong study which included children and adult patients with chronic rhinitis reported that 14% had cat allergy [69]. A multicenter study in China among patients with asthma and/or rhinitis found prevalence of 10.3% cat allergy [23].

We found cat allergy was significantly related to wheeze, wheeze when not have a cold, at least one airway symptoms, doctor-diagnosed asthma, current asthma and nasal allergies medication, even after mutual adjustment in statistical tests. Moreover, cat allergy was an independent risk factor for respiratory symptoms of the respondents, even after adjusting for HDM allergy and other potential confounders in this study. This result supports the finding of previous research that cat sensitization is an important risk factor for asthma in adults [57,70]. One limitation of our study is that we have no data on the cat allergen level in the office environment. In a community with high levels of cat ownership, people who are allergic to cats may get exposed to high levels of cat allergen, even though they do not own a cat in their home, since cat allergens can be passively transported with ease through clothing [71]. Our results indicate a clear need to further study the prevalence of cat allergy and its health implication in Malaysia as well as in other Asian countries.

HDM allergy was very common in our study and the majority (52.5%) of the office workers were sensitized to HDM allergens (*D*. *pteronyssinus* or *D*. *farinae*). Most (46.9%) were sensitized to both allergens, only 2.8% and 1.9% were sensitized to one HDM allergen only (only *D*. *pteronyssinus* or only *D*. *farinae*). We did not find any previous population based study of HDM allergy among adults in Malaysia. There is a limited number of population based studies investigating HDM allergy among adults in other countries in Asia. One population based study from Singapore found that 68.5% of adults were sensitized to *D*. *pteronyssinus* [72]. Another population study from Thailand found that 38% of adult asthmatics and 25% of adults without asthma were sensitized to HDM allergens [21]. One population based study from the northern part of Vietnam reported lower prevalence of HDM allergy among adults, 13.3% allergy to *D*. *pteronyssinus* and 10.5% allergy to *D*. *farinae* [22]. One population study from Fukui prefecture and central Hokuriku area of Japan found that 50% of all adults were sensitized to HDM allergens [73]. The ECRHS II, a population study performed in 10 different countries (mainly in Western Europe) reported that 14.1% of adults were sensitized to HDM [64]. Thus, the high prevalence of HDM allergy in our study is in agreement with most population studies from Asia.

Most studies on HDM allergy in Asia had been performed among hospital patients with asthma or allergic rhinitis [21,53,67,68,74–77]. We found five patient studies of HDM allergy in Malaysia, mostly including both children and adults. One study reported 80.0% HDM allergy among adult patients [78]. Another study found 63.3% HDM allergy by skin prick test among adult patients [4]. Another study (age range 6–60 years) reported 82% HDM allergy [5]. Another study (age range 12–76 years) found 81.4% allergy to *D*. *farinae* and 93.6% allergy to *D*. *pteronyssinus* [6]. Finally, one recent study (age range 5–64 years) found 69.7% of HDM allergy [7]. One large multicenter study among allergy patients at different hospitals in China observed the prevalence of HDM allergy increased from north to south and with increasing temperature and RH [23]. Warm and humid climate facilitate HDM growth and can explain the high prevalence of HDM allergy found in this study as well as other studies in parts of Asia with similar climate.

Our findings concerning the health associations for HDM allergy are in agreement with many previous early studies, reporting that HDM allergy and exposure to HDM allergens were associated with asthma [79–81], asthma symptoms [82–86], and rhinitis [22]. HDM allergens can cause allergy, asthma and allergic airway inflammation by inducing adaptive Th2-based adaptive immune responses [87] and also activate innate immune cells [88,89].

The major site of exposure to HDM allergens is in the home environment, since HDM mainly live in bed mattresses [1,28,90]. Home environment studies from Malaysia reported high prevalence and density of *B*. *tropicalis* (average 8,934 mites/g per month), *D*. *pteronyssinus* (average 4,553 mites/g per month) and *M*. *intermedius* (average 1,020 mites/g per month) [2]. The allergens might be transported from the home by cloths. Neal and colleagues found live and dead mites in automobiles and clothing [91]. Nevertheless, the mites can also multiply or colonize in carpets or upholstered furniture if provided with sufficient food, protection, warmth and moisture [2,26]. One recent study on allergens in school dust in Malaysia found very low levels of house dust mites Der p 1 (range 2–33 ng/g); Der f 1 (range 4–50 ng/g), as well as low levels of the tropical storage mite Blo t (range 0.6–5.3 AU/g) [3]. There are few studies on HDM allergens in office dust. The levels of HDM allergens in office dust in our study was lower than the HDM allergen levels measured in the large United States office study [26] but higher than the HDM allergen levels measured in an Italian office study [27]. The levels in our study were comparable with the levels from another office study performed in Nanjing, China and United States [90].

To our knowledge, our study is the first study measuring HDM allergen levels in offices in Malaysia. We found positive associations between Der f 1 level and daytime breathlessness especially in subjects allergic to HDM. Moreover, we found higher levels of Der f 1 as compared to Der p 1 in office dust. Most other studies from the Asia-Pacific region were performed in homes, and have reported higher levels of Der p 1 level as compared to Der f 1 [92]. However, two other office studies found higher levels of Der f 1 level as compared to Der p 1 [26,28]. This might be because the dry indoor climate of the mechanical ventilated and air conditioned offices promoted the growth of *D*. *farinae*. There are biological and ecological differences between *D*. *pteronyssinus* and *D*. *farinae*. *D*. *farinae* is able to survive better than *D*. *pteronyssinus* in drier areas [93,94]. In our study, negative associations between Der p 1 level with wheeze and at least one airway symptoms were found. However, it cannot be interpreted as Der p 1 has protective effect on wheeze and airway symptoms because Der p 1 allergen involve in mechanisms (modulate differentiation and maturation of dendritic cells) which leading to Th2 cytokine immune response [89]. This negative association might be due to residual confounding since it was most pronounced in non-allergic subjects.

In conclusion, in our study HDM allergy, cat allergy and respiratory symptoms and rhinitis were very common among office workers in Malaysia. Moreover, HDM allergy and cat allergy were independently associated with asthma, airway symptoms and rhinitis. Further studies on allergy among office workers and other population studies in Malaysia should include allergy testing against HDM and cat allergy since these two types of allergens seem to be major allergens in this country. However, the health relevance of other less common IgE-mediated allergies in Malaysia also need to be further investigated. In office environments in Malaysia, Der f 1 allergens can be a risk factor for daytime respiratory symptoms among office workers.
